# A Digital Platform to Support Self-management of Multiple Chronic Conditions (ProACT): Findings in Relation to Engagement During a One-Year Proof-of-Concept Trial

**DOI:** 10.2196/22672

**Published:** 2021-12-15

**Authors:** Julie Doyle, Emma Murphy, Shane Gavin, Alessandra Pascale, Stéphane Deparis, Pierpaolo Tommasi, Suzanne Smith, Caoimhe Hannigan, Myriam Sillevis Smitt, Cora van Leeuwen, Julia Lastra, Mary Galvin, Patricia McAleer, Lorraine Tompkins, An Jacobs, Marta M Marques, Jaime Medina Maestro, Gordon Boyle, John Dinsmore

**Affiliations:** 1 NetwellCASALA Dundalk Institute of Technology Dundalk Ireland; 2 Trinity Centre for Practice and Healthcare Innovation School of Nursing and Midwifery Trinity College Dublin Dublin Ireland; 3 TU Dublin Dublin Ireland; 4 IBM Research Europe Dublin Ireland; 5 Department of Psychology National University of Ireland, Dublin Dublin Ireland; 6 Imec-VUB-SMIT Brussels Belgium; 7 Tree Technology Madrid Spain; 8 ADAPT SFI Research Centre Trinity College Dublin Dublin Ireland

**Keywords:** digital health, aging, multimorbidity, chronic disease, self-management, integrated care, longitudinal study, engagement, usability, mobile phone

## Abstract

**Background:**

Populations globally are ageing, resulting in higher incidence rates of chronic diseases. Digital health platforms, designed to support those with chronic conditions to self-manage at home, offer a promising solution to help people monitor their conditions and lifestyle, maintain good health, and reduce unscheduled clinical visits. However, despite high prevalence rates of multimorbidity or multiple chronic conditions, most platforms tend to focus on a single disease. A further challenge is that despite the importance of users actively engaging with such systems, little research has explored engagement.

**Objective:**

The objectives of this study are to design and develop a digital health platform, ProACT, for facilitating older adults self-managing multimorbidity, with support from their care network, and evaluate end user engagement and experiences with this platform through a 12-month trial.

**Methods:**

The ProACT digital health platform is presented in this paper. The platform was evaluated in a year-long proof-of-concept action research trial with 120 older persons with multimorbidity in Ireland and Belgium. Alongside the technology, participants had access to a clinical triage service responding to symptom alerts and a technical helpdesk. Interactions with the platform during the trial were logged to determine engagement. Semistructured interviews were conducted with participants and analyzed using inductive thematic analysis, whereas usability and user burden were examined using validated questionnaires.

**Results:**

This paper presents the ProACT platform and its components, along with findings on engagement with the platform and its usability. Of the 120 participants who participated, 24 (20%) withdrew before the end of the study, whereas 3 (2.5%) died. The remaining 93 participants actively used the platform until the end of the trial, on average, taking 2 or 3 health readings daily over the course of the trial in Ireland and Belgium, respectively. The participants reported ProACT to be usable and of low burden. Findings from interviews revealed that participants experienced multiple benefits as a result of using ProACT, including improved self-management, health, and well-being and support from the triage service. For those who withdrew, barriers to engagement were poor health and frustration when technology, in particular sensing devices, did not work as expected.

**Conclusions:**

This is the first study to present findings from a longitudinal study of older adults using digital health technology to self-manage multimorbidity. Our findings show that older adults sustained engagement with the technology and found it usable. Potential reasons for these results include a strong focus on user-centered design and engagement throughout the project lifecycle, resulting in a platform that meets user needs, as well as the integration of behavior change techniques and personal analytics into the platform. The provision of triage and technical support services alongside the platform during the trial were also important facilitators of engagement.

**International Registered Report Identifier (IRRID):**

RR2-10.2196/22125

## Introduction

### Background

Multimorbidity, the presence of 2 or more chronic conditions in an individual [1], is a major global health concern [2], and in many high-income countries, it is now considered the norm rather than the exception [3]. Some evidence suggests that it is more common in older adults, likely because of the aging population, whereas other research states that it is more prevalent in disadvantaged groups, such as those with lower socioeconomic status [3,4]. Prevalence rates suggest that, globally, 1 in 3 people live with multimorbidity, with rates of 65% in people aged >65 years and 85% in people aged >85 years and rising [5]. However, multimorbidity is also known to affect younger adults, with social deprivation being a key determinant in younger and middle-aged adults [3,6]. Multimorbidity reduces life expectancy, decreases quality of life and physical functioning, and negatively affects mental health [7]. It also results in health inequalities [4]. Some evidence suggests that reported outcomes, such as lower self-reported health, increased medication issues, and higher health care utilization, are poorer for older populations [8].

Self-management is recognized as an important component of care for those with multimorbidity to maintain good health [9,10]. Self-management can be defined as the actions taken by an individual to manage symptoms, treatment, emotions, and lifestyle changes as part of living with a chronic condition [11]*.* Compared with those with one chronic condition, people with multimorbidity experience more challenges with self-management, associated with numerous self-care tasks such as symptom monitoring, management of multiple medications, and liaising with multiple health care professionals [10,12-14]. For people with multimorbidity, self-management is an iterative process requiring constant readjusting and reframing to understand their conditions and the associated changeable symptoms to inform personalized self-management responses [15,16].

Numerous digital health technologies have been developed to support the self-management of single chronic diseases, primarily diabetes [17]. However, given the rise in the number of people managing multiple chronic conditions, it is imperative to consider how such technologies can be designed and implemented to deal with the additional complexities of multimorbidity, such as the management of multiple symptoms and self-care tasks. Prior work has noted that technologies that help those with multimorbidity manage their health will only be successful if they do not exert further burden or inconvenience on the user [18]. Furthermore, little attention has been directed toward the importance of understanding how to support people with multimorbidity through the use of digital interventions [19]. Integrating the management of multiple conditions onto a single platform, where users can monitor their symptoms and relevant lifestyle parameters (such as activity), interact with all their data, share their data, and receive educational support, could help to minimize the known burden of multimorbidity self-management. In recent years, a small number of researchers have begun to examine how to design digital health apps for multimorbidity self-management [20-27], including medication management [20,28], how to manage health care conflicts [21,24], and how those with multimorbidity collaborate and communicate with informal caregivers and health care professionals [25,27]. However, we are unaware of platforms that have been implemented to tackle multimorbidity or evaluated over longitudinal periods. Indeed, a recent systematic review has highlighted the lack of digital health platforms, that include architectures and analytics capable of fully supporting chronic disease self-management [29], beyond what is possible through simple *apps*. The review identified 7 papers, all of which support single disease management, and not multiple diseases.

The research on longitudinal engagement with digital health and wellness technologies, particularly for older adults, is lacking. Engagement is necessary to achieve intended and effective outcomes [30]. However, it is understood that the adoption of digital health technologies is low [18]. It has also been argued that older adults are not seen as the primary users of such technology [31] and are not ready to adopt it [32]. However, recent research goes some way to resolve this issue. A study examining the use patterns of 9051 users (mean age 50.4 years) with a diabetes management app over a period of 180 days found that older adults were more actively engaged with the app than younger users [33]. Wei et al [34] studied habit formation using wearable activity trackers with 20 older adults who had been tracking activity for 6 months. They found a range of factors contributing to sustained use, such as contextual factors such as the placement of the tracker, how often it needs to be charged, and the presence of features such as goal setting and reminders. It is generally accepted that the successful design of a health and well-being intervention to motivate engagement requires a user-centered design process and an iterative approach to development [29,30]. This is particularly important for older adults, who may be unfamiliar with such technology and who may have additional interaction requirements because of the physical and cognitive effects of aging [31]. It is therefore crucial that designers of health technologies for older adults involve them closely in the design of technologies to ensure that they can benefit from them.

Our main objectives were as follows:

1. Design and develop a digital health platform (ProACT) to support people with multimorbidity to self-manage multiple conditions on a single platform, including monitoring a collection of symptoms and well-being parameters, helping users to understand relationships between their symptoms and conditions, providing education personalized to the person’s condition profile, and supporting the sharing of data with a care network.
2. Evaluate ProACT in a year-long proof-of-concept (PoC) trial with people with multimorbidity supported by people in their care network to determine engagement, usability, and experiences with the digital self-management of a set of multiple symptoms and well-being parameters.

In this study, we present the ProACT platform that is designed to support people with multimorbidity to self-manage multiple conditions and results from a 12-month PoC trial of the platform with respect to engagement and usability. Our findings indicate that the vast majority of people with multimorbidity stayed engaged with the platform over the 12-month period, a novel finding with respect to this cohort. The participants found the platform to be usable and of low burden. Qualitative data from participant interviews indicated that participants experienced a number of benefits as a result of using ProACT, such as improved self-management, health, and well-being. For those who withdrew, barriers to engagement were poor health and frustration with the elements of the technology, particularly the sensing devices to monitor health parameters.

### The ProACT Platform

The aim of the ProACT project was to design a single platform for supporting people with multiple conditions, specifically 2 or more of the following: chronic obstructive pulmonary disease (COPD), congestive heart failure (CHF), chronic heart disease (CHD), and diabetes, to self-manage. These conditions were chosen, as the World Health Organization identified the 4 key types of chronic diseases as cardiovascular, chronic respiratory diseases, diabetes, and cancer [35]. In addition, outcomes from a multimorbidity study by Barnett et al [6], with data collected from 1,751,841 people in 314 medical practices in Scotland, showed CHF, COPD, and diabetes as significantly linked disease conditions with related comorbidities (eg, hypertension). Although the platform was initially developed to support people self-managing combinations of these conditions, it is sufficiently flexible to allow new conditions to be added to the platform over time.

**Figure 1:**
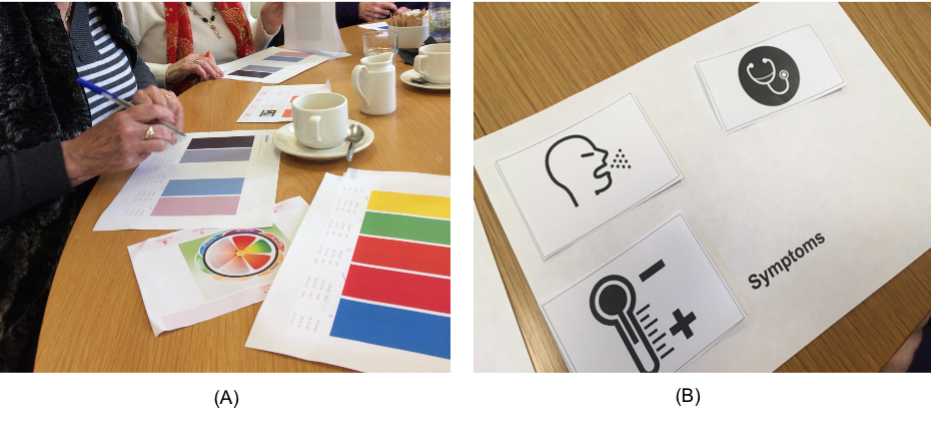
Participants in co-design workshops (A) choosing color schemes; (B) matching icons to labels.

The ProACT platform was designed and developed using an extensive user-centered design process. This process involved interviews, focus groups, co-design sessions (hands-on design activities with participants; Figure 1), and usability testing before the platform’s deployment in the trial. A total of 58 people with multimorbidity and 106 care network participants, including informal carers, formal carers, and health care professionals, across Ireland and Belgium participated in this process. The findings from the user-centered design process have been published elsewhere [21,28,36,37].

The ProACT platform, designed and developed based on the findings from the above phases, has the following components:

- Measurement and sensing devices: off-the-shelf devices were used to collect health (blood pressure, heart rate, blood glucose, and pulse oximetry) and well-being (weight, activity, and sleep) readings from people with multimorbidity in their homes.
- ProACT person with multimorbidity CareApp: a web-based app delivered on an interactive device such as a tablet or smartphone that supports the monitoring of person with multimorbidity status and provides feedback and education to support health and wellness self-management. The CareApp has been designed to minimize the burden of self-management, for example, by helping people with multimorbidity prioritize condition management.
- ProACT care network CareApps: customized CareApp interfaces, developed for people in the person with multimorbidity’s care network (informal carers, formal carers, and health care professionals), enables the viewing of the person with multimorbidity’s health and well-being data, once the person with multimorbidity has agreed to share it.
- Context-aware brokering and inference engine (CABIE+): a source-agnostic data collection system to collect and organize sensor and self-reported data collected through the CareApp.
- Subject information management system (SIMS): a user management system allowed researchers or service organizations to personalize CareApps to individuals and manage, inspect, and analyze data.
- SIMS Triage: a version of the SIMS user management system was developed specifically for the clinical triage staff to view and respond to risk-stratified alerts from data collected by people with multimorbidity. This system provides clinical triage staff with a holistic view of the person with multimorbidity, displaying data relevant to their various conditions.
- KITE (Knowledge InTEgration) platform: a cloud-based infrastructure that allows the aggregation and processing of health data using a dynamic set of analytical components.
- CareAnalytics: innovative analytics to detect and react to data collected using ProACT. CareAnalytics support multimorbidity self-management, for example, by ensuring that any recommended content considers the person’s overall condition profile, and behavior change, for example, by recommending educational content or highlighting a condition that needs attention, based on a person with multimorbidity’s current health and well-being status.

**Figure 2:**
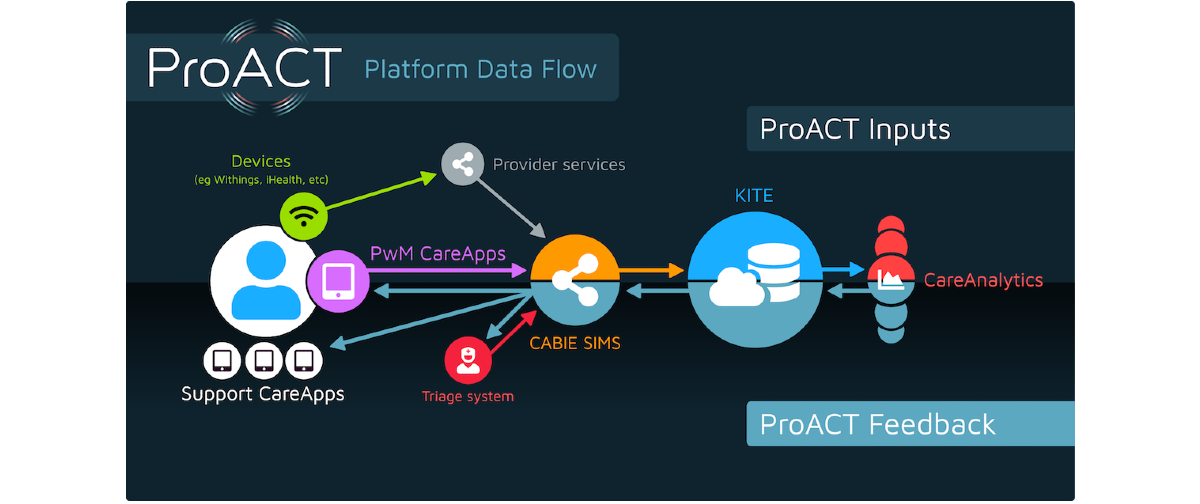
Data flow within the ProACT platform. CABIE: context-aware brokering and inference engine; KITE: Knowledge InTEgration; PwM: person with multimorbidity; SIMS: subject information management system.

The platform focused primarily on supporting the management of a collection of health symptoms and well-being parameters (relevant to the conditions of interest outlined above), helping users to understand the relationships between their symptoms and conditions, providing education relevant to individual conditions while also ensuring that this education considers multiple conditions, and provides a single app where people with multimorbidity or care network users could view all data and relevant content. The platform was developed to be compliant with the European Union’s (EU’s) General Data Protection Regulation (Regulation [EU] 2016/679) and underwent a data protection impact assessment before its deployment in the trial. Figure 2 illustrates the data flow between the different ProACT components. The following section provides a more in-depth overview of the components, primarily focusing on the person with multimorbidity CareApp.

### ProACT Person With Multimorbidity CareApp

#### Overview

In this section, we describe the CareApp used by the person with multimorbidity participants during the PoC trial. Further details on how we mapped specific user requirements to ProACT design features have been published elsewhere [37]. The following subsections outline the various features within the CareApp. The content of the CareApp is personalized at both *condition* and individual levels. For example, participants with diabetes and COPD will not see any features or content with respect to CHF management. Individuals with diabetes may decide that they do not want to collect blood glucose data. This personalization is achieved within the SIMS system and is described below. The CareApp was developed as a responsive web app and is therefore accessible across a wide range of devices and platforms.

#### Symptom Monitoring and Reflection

**Figure 3:**
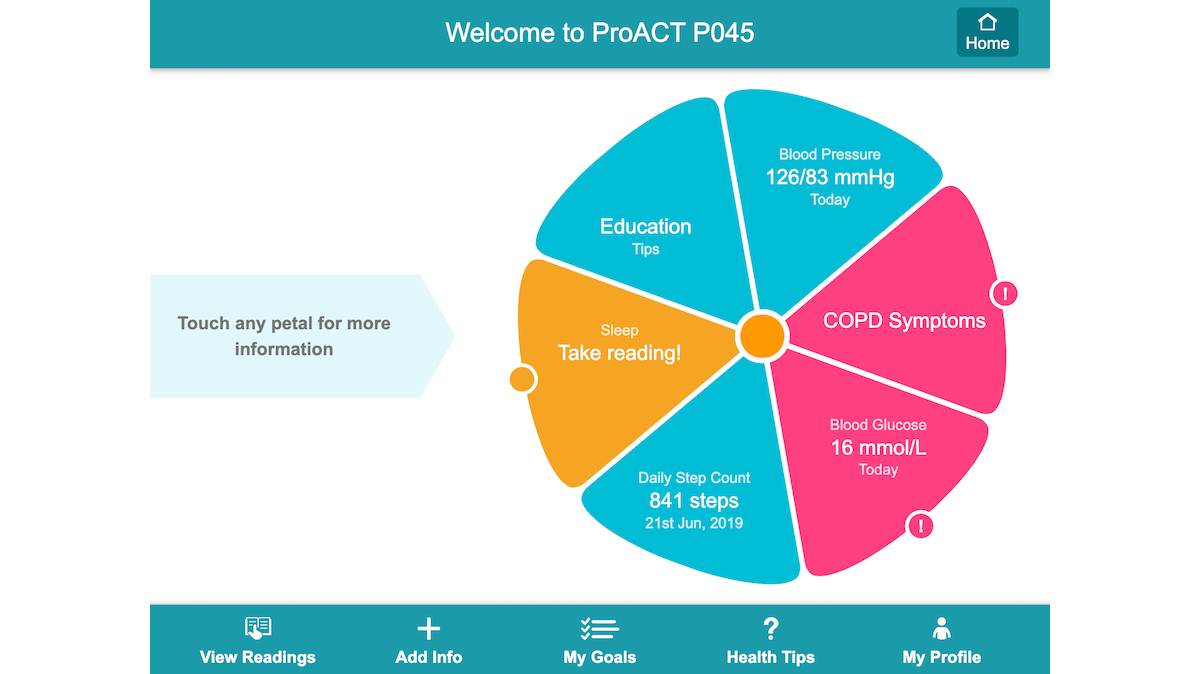
CareApp dashboard.

**Figure 4:**
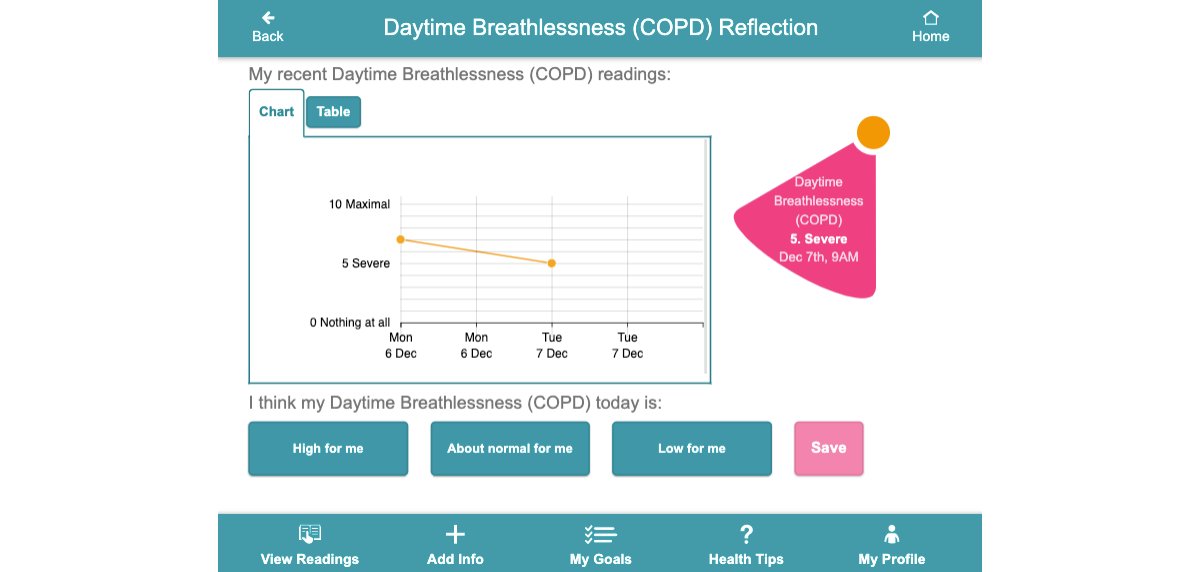
Reflection screen for abnormal chronic obstructive pulmonary disease symptoms.

**Figure 5:**
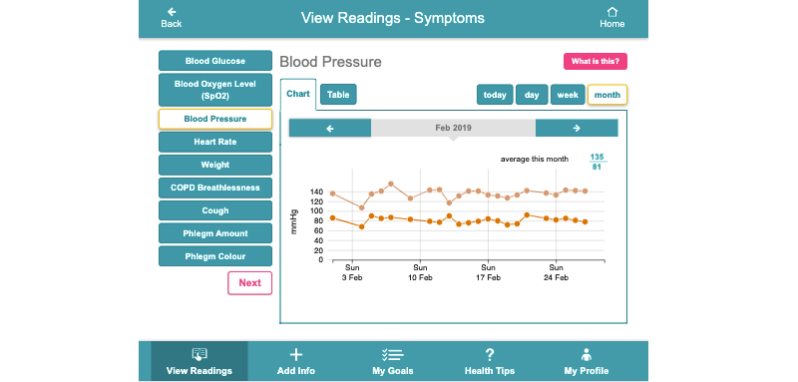
Data trends over time.

Findings from our requirements gathering study highlighted that those managing multimorbidity need to monitor several health and well-being parameters on a regular basis; therefore, digital health apps to support multimorbidity need to consolidate multiple health and well-being parameters into a single app [21]. Access to data has also been shown to support behavioral change and the self-management of chronic conditions [16]. Where available, off-the-shelf sensors and devices were sourced to monitor these parameters, whereas questions delivered through CareApp’s *Add Info* section supported the monitoring of additional parameters not measured through a device (eg, foot care for diabetes, fatigue, and breathlessness). Figures 3-5 show the person with multimorbidity CareApp. The flower of the dashboard (Figure 3) provides a quick overview of the person with multimorbidity’s current status (eg, their current step count and their last blood pressure reading). Petals can be blue, orange, or pink. An orange petal highlights to the user that they have not taken a reading for a particular parameter in the past 5 days. Pink represents a *nudge* to the user to further explore the petal, for example if a reading is outside the person’s defined *normal* threshold. A symptom reflection feature was designed as an extension of the flower dashboard design. Figure 4 shows what happens when a user clicks on the pink petal containing COPD symptoms in Figure 3. In addition to measuring and viewing their symptoms, this feature encourages users to *reflect* on their recent symptom readings with respect to their normal readings by asking them whether their reading is within a normal range for them. This feature was designed to support people in understanding their symptom readings rather than passively recording them. This may be particularly important when a person first starts self-managing with the platform, whereas later reflection may become more intrinsic.

Findings from our requirements study revealed that, sometimes, the management of one chronic condition can be forgotten, particularly if another is currently more acute [21]. The flower design, and the logic behind it, ensures that if a condition is not being monitored, it is brought to the attention of the person with multimorbidity. This could be a prompt or alert to monitor symptoms relating to that condition or educational content being pushed to them. The flower acts as a subtle, unobtrusive prompt—it is up to the person with multimorbidity to act on it. Our requirements study [21] and research by others [12,38] have identified that with multiple self-management tasks across different conditions, there is an increased need to support people with multimorbidity in prioritizing their activities, to reduce complexity and time burden. Therefore, within the CareApp, only the areas that require attention are highlighted. In addition to the flower petals, people with multimorbidity can view any of their historical readings through the *View Readings* section of the CareApp (Figure 5).

#### Education and Training

**Figure 6:**
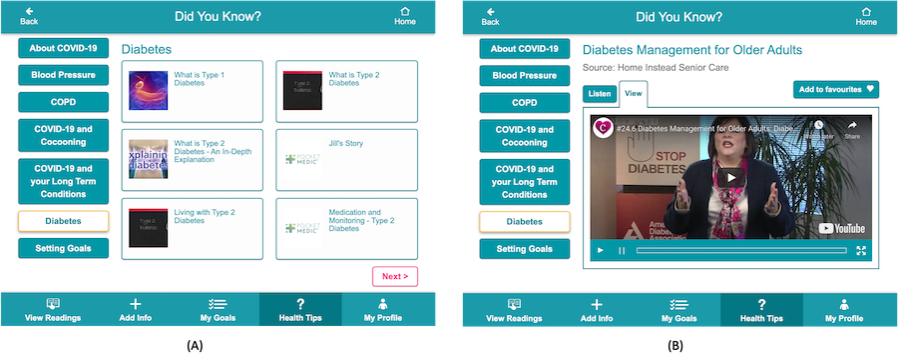
Educational content in ProACT (A)The library of educational content; (B) A tip in video format.

Findings from the requirements study, and those of others, highlighted that a lack of information is a significant barrier to both effective self-management and motivation to engage in self-management actions [21,39,40]. The selection of content for the education section of the CareApp and its planned delivery were therefore important tasks, and it was important to ensure the provision of trusted, reliable information tailored to a person with multimorbidity’s specific conditions and management needs (including device and CareApp training). Within the *Health Tips* section of the CareApp, there are two categories of content: *Did you know?* contains educational content relevant to self-management of the person’s conditions and well-being (Figure 6), and *How do I?* contains custom-made video training content on how to use the devices and CareApp. Educational information for each disease was sourced from reputable sources known to people with multimorbidity (eg, from national health services in each country). Where possible, content was delivered in three modalities (video, audio, and text) to cater to differences in learning styles and accessibility. An important consideration for the management of multiple conditions is that any education provided to the person with multimorbidity considers their current health status and their complete condition profile. This is achieved through ProACT CareAnalytics, as discussed in the section *CareAnalytics*.

#### Personal Goals

Setting goals and progressing toward goal achievement are key features for supporting the self-management of multimorbidity. People with multimorbidity can set physical activity goals in the ProACT CareApp. An overview of their weekly progress, measured through their activity watch, and an intuitive interface to help them set different goal modalities (eg, steps, distance, and time spent walking), are accessible through the *My Goals* section of the CareApp. Through messages and prompts, ProACT users are supported in setting achievable and incremental goals. The goal recommender described in the *CareAnalytics* section below suggests realistic activity goals based on the user’s most recent activity data. As the user progresses and surpasses their targets, prompts suggest more challenging goals. Similarly, if the person with multimorbidity has difficulty reaching their target, they can say why this is by choosing a reason from a predefined list (eg, they were unwell this week), providing some context for why a particular goal was not achieved. Our requirements gathering identified additional features with respect to goal setting, such as collaborative goal setting with the person with multimorbidity’s care network and feedback on goal progress [36], which will be integrated into future versions of the CareApp.

#### The Care Network

People with multimorbidity should be empowered to be contributors in the selection of their care network. Research has shown that the effective self-management of chronic disease does not occur in isolation but often involves, and is directly influenced by, support from informal carers and formal carers, as well as health care professionals during clinical visits [41,42]. Our findings from the requirements gathering also highlight, however, that the person with multimorbidity is often the coordinator of their own care, given the lack of integration among health care providers. Therefore, it is important that the person with multimorbidity can choose whom, within their care network, can support and contribute to their digital self-management. A feature within the *My Profile* section of the person with multimorbidity CareApp supports this, whereby the person with multimorbidity can add someone to their network and choose what data to share with them. CareApps for informal carers, formal carers, and health care professionals are accessible on their own devices (all CareApps are web based and responsive), allowing these care network stakeholders to see person with multimorbidity data that have been shared with them.

#### Usability and Accessibility

The usability and accessibility of the technology are crucial to ensure that users can easily interact with it. The CareApp was designed and developed to comply with relevant accessibility guidelines from tools, which are as follows:

- The Web Content Accessibility Guidelines 2.0 [43]: these guidelines state that (1) content must be perceivable; (2) interface components in the content must be operable; (3) content and controls must be understandable; and (4) content should be robust enough to work with current and future user agents (including assistive technologies).
- British Broadcasting Corporation mobile accessibility guidelines [44]: the British Broadcasting Corporation standards and guidelines for mobile accessibility are a set of technology-agnostic best practices for mobile web content and hybrid and native apps.
- Automated accessibility checkers and tools: although not as accurate as manual audits, automated accessibility checking tools such as the Web Content Accessibility Guidelines 2.0 offer better accuracy. A checker compliance tool (website shutting down in April 2021 [45]) was used. In addition, as color and contrast are important accessibility features for older users, tools were sourced that automatically check that colors have sufficient contrast within the interface (such as the web aim color contrast checker; [46]).

An accessibility and traceability spreadsheet was maintained detailing each guideline and was used to record accessibility issues and outline how they were resolved during the design of the CareApps. Accessibility guidelines should only form a part of an inclusive user-centered design process, as the most definitive test of accessibility can only be evaluated with end users [47]. End users, including people with multimorbidity and care network stakeholders, were involved as co-designers throughout the entire iterative design and development process. Usability sessions were conducted with person with multimorbidity users from the early design phases, and throughout the various time points of the trial, allowing us to observe and gather feedback on usability and accessibility. The results of these evaluations informed interface updates, further enhancing the usability and accessibility of the app iteratively across the project.

### Context-Aware Brokering and Inference Engine

CABIE+ is a source-agnostic cloud-based data aggregation platform. CABIE+ was the primary exchange mechanism used to connect the distinct technology components in ProACT. This component was used to centrally collect data from all connected devices used by people with multimorbidity, to normalize these data for storage, to make these data available to additional CABIE+ and ProACT components (SIMS and KITE), and to provide a configurable processing pipeline allowing incoming data to be inspected and reacted to in real time.

### Subject Information Management System

SIMS is an administrative tool that facilitates the management of trial technologies, provides an abstraction layer for managing multiple CABIE+ instances and provides researchers or service organizations with a user-friendly, centralized service for monitoring and inspecting the various elements of the ProACT platform. SIMS also provides a user-facing application programming interface, which was used in the creation of the ProACT CareApps.

**Figure 7:**
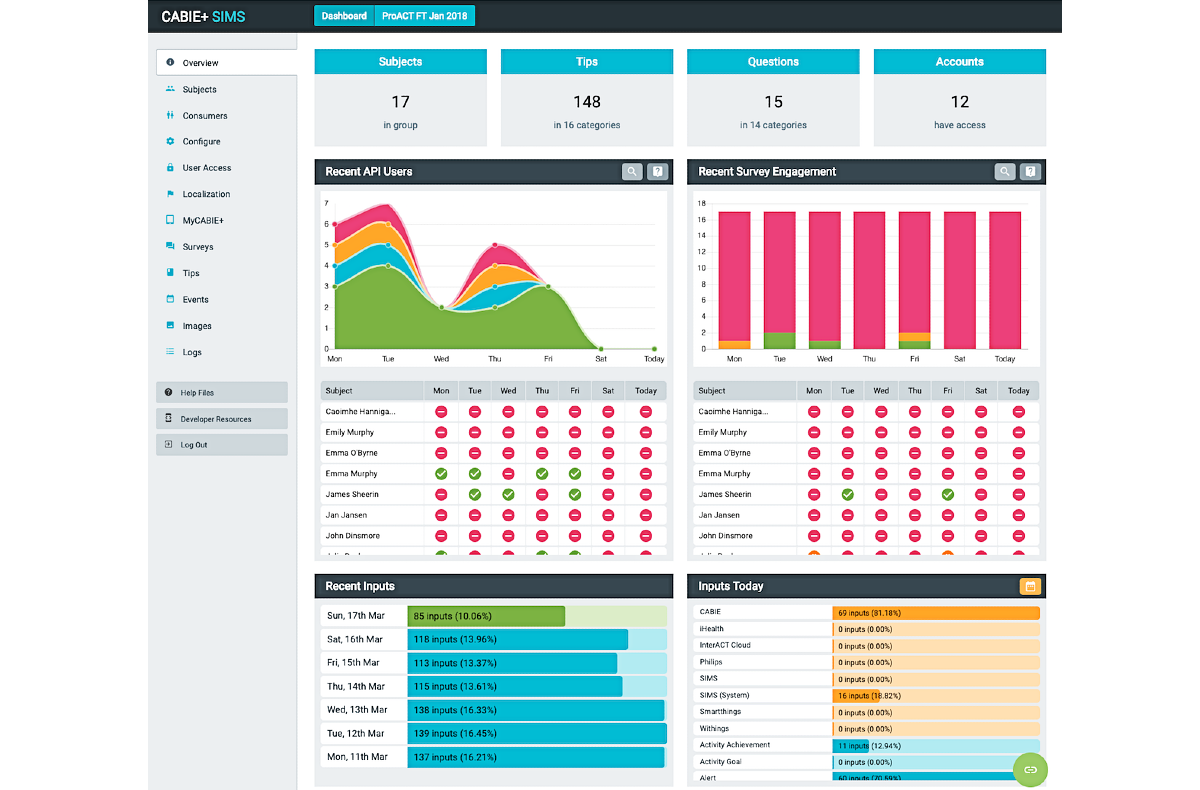
Subject information management system dashboard, which allows for personalization and configuration of CareApps for person with multimorbidity and inspection of their data (participant names removed from image).

**Figure 8:**
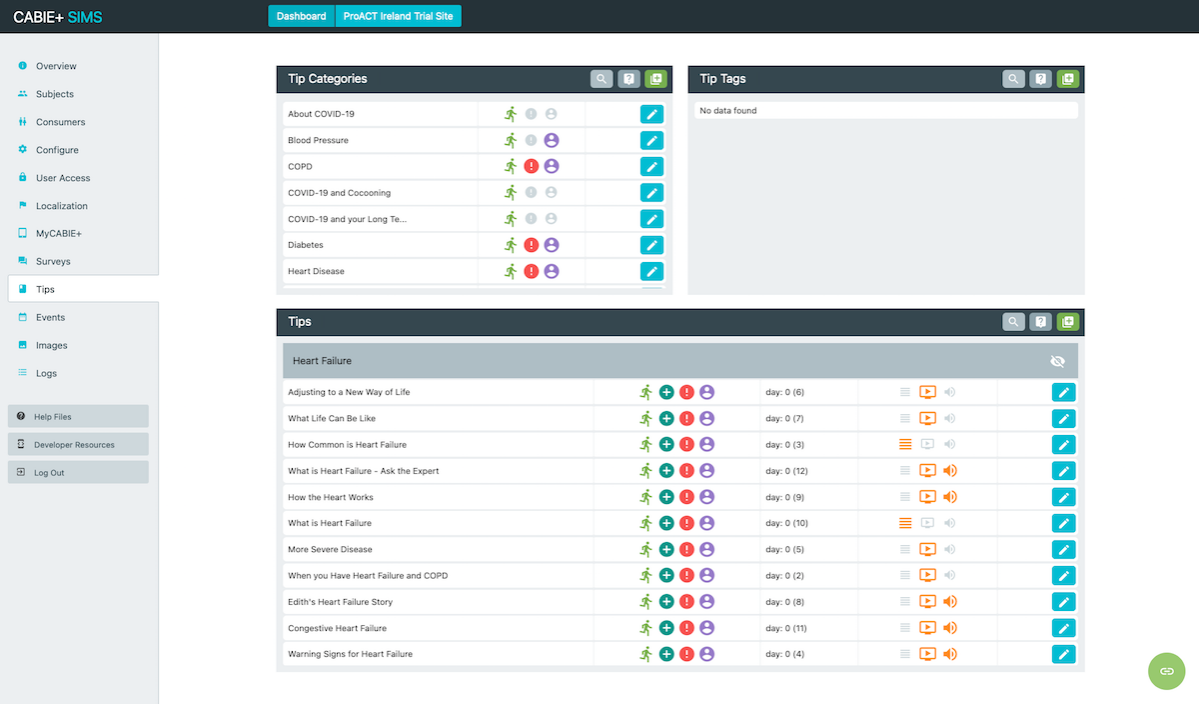
Subject information management system interface showing tip categories available and a number of tips related to heart failure.

**Figure 9:**
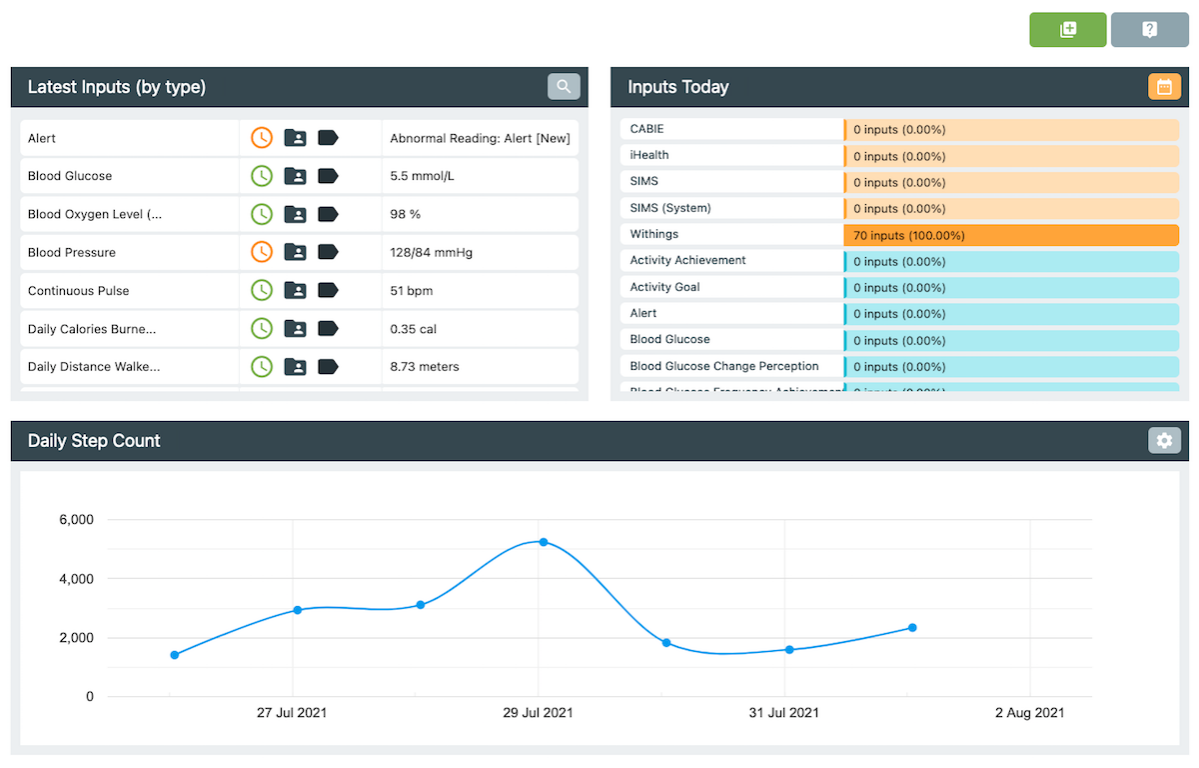
Inspect feature in subject information management system allows querying of data for each participant and shows their most recent inputs.

SIMS provides a number of features to facilitate the setup and management of trials and data. End users can be added to the system and their personalized profiles can be configured; for example, entering demographic information, configuring conditions being monitored within ProACT, and linking sensors and devices to their profile (Figure 7). Adding and scheduling educational content and self-report questions for delivery through the CareApp was also done via SIMS (Figure 8). For example, various categories of education and self-report questions can be created and delivered to all participants or to those with specific conditions. Furthermore, only participants configured in the system as having heart failure will see educational content and symptom questions related to that condition. Participant data can be queried via the *Inspect* feature (Figure 9), which shows the most recent inputs for each participant, including how long ago they logged in to the system (green indicates within the past day, red over a week ago). Each parameter measured by the participant can also be queried and viewed as a chart or table or exported for further analysis. SIMS also allows for personalized symptom thresholds to be set and generates alerts when symptom readings fall outside these thresholds (eg, alerting a triage nurse when a person’s blood glucose level is outside their normal parameters).

**Figure 10:**
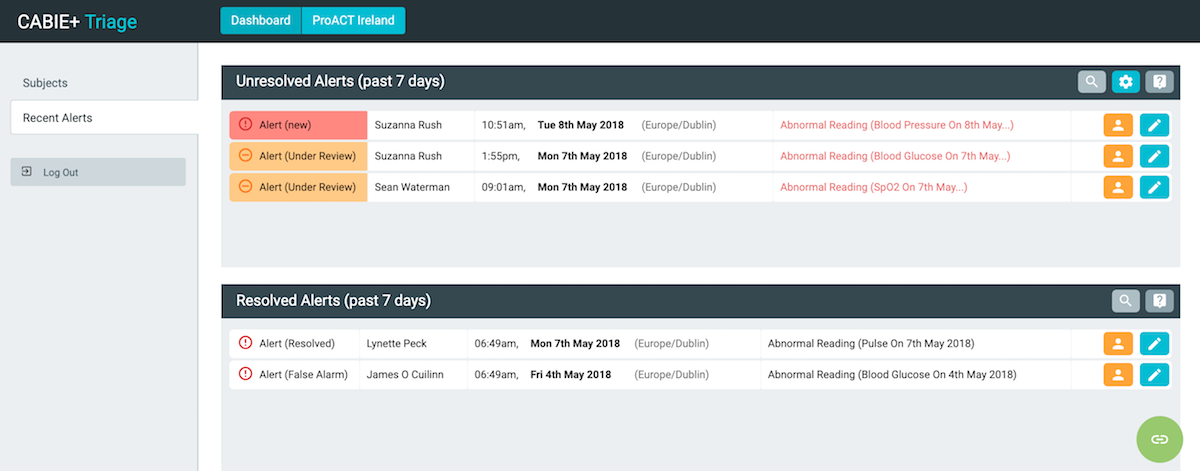
Subject information management system triage interface dashboard showing alerts (real names are not used).

The SIMS triage interface (Figure 10) presents clinical triage nurses with a list of alerts for individuals. Alerts are generated when thresholds for different parameters, defined within the system, are breached. For example, an alert for high blood glucose is a reading over 14 mmol/L (configurable per participant). Participants’ placement on the list is prioritized by their alert status, that is, those with a *red alert* status appear first. A tag also appears alongside the alert, indicating whether it is new or under review. Within the dashboard, the nurse can also view recently resolved alerts. Nurses can query the person with multimorbidity’s health and well-being data (as shown in Figure 9) to give them a holistic picture of the person with multimorbidity’s status before calling them to discuss their alerts. Nurses can also create notes with respect to alerts, thereby allowing for a rich description of the context linked to alert readings.

### Knowledge InTEgration

**Figure 11:**
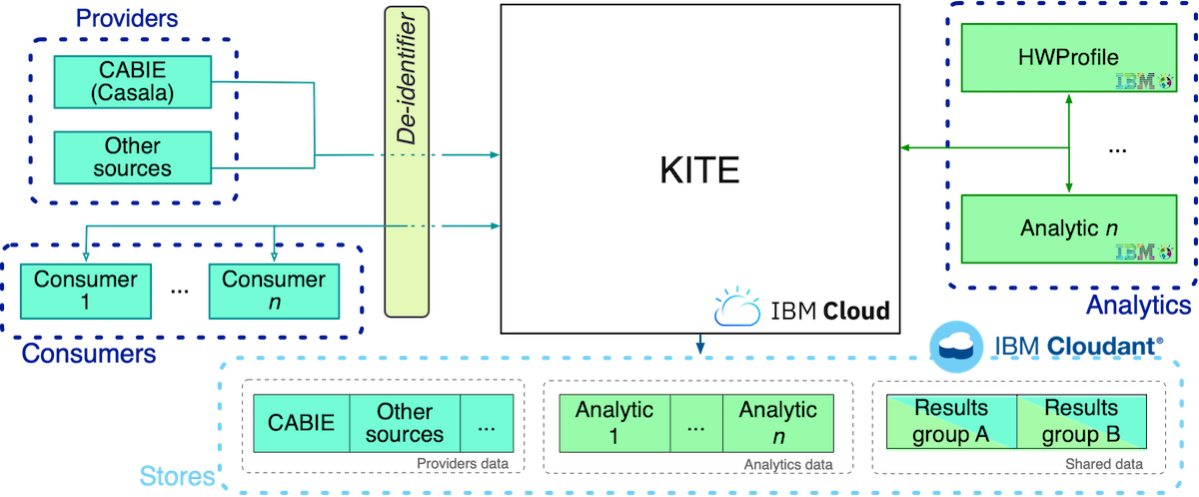
High-level Knowledge InTEgration architecture. KITE: Knowledge InTEgration.

KITE is a cloud-based platform providing a reliable storage system for large volumes of data. In addition to being highly scalable, it eases data maintenance and access through an easily configurable data access configuration and transparently kept traceability metadata. KITE is exposed as a set of authenticated services for managing deidentified health data and coordinating collaboration among data providers, system data analytics, and data consumers. Figure 11 shows the actors of a typical workflow within KITE: providers push new deidentified data (eg, sensor data, self-reports, questionnaire results), which read the already deidentified data and create new results, whereas consumers read analytics’ output and show it to the final users (people with multimorbidity and care network members). In a real-world scenario, analytics can selectively share output among themselves, allowing a complex CareAnalytic to be decomposed into a set of simpler and smaller analytics before reaching a data consumer.

**Figure 12:**
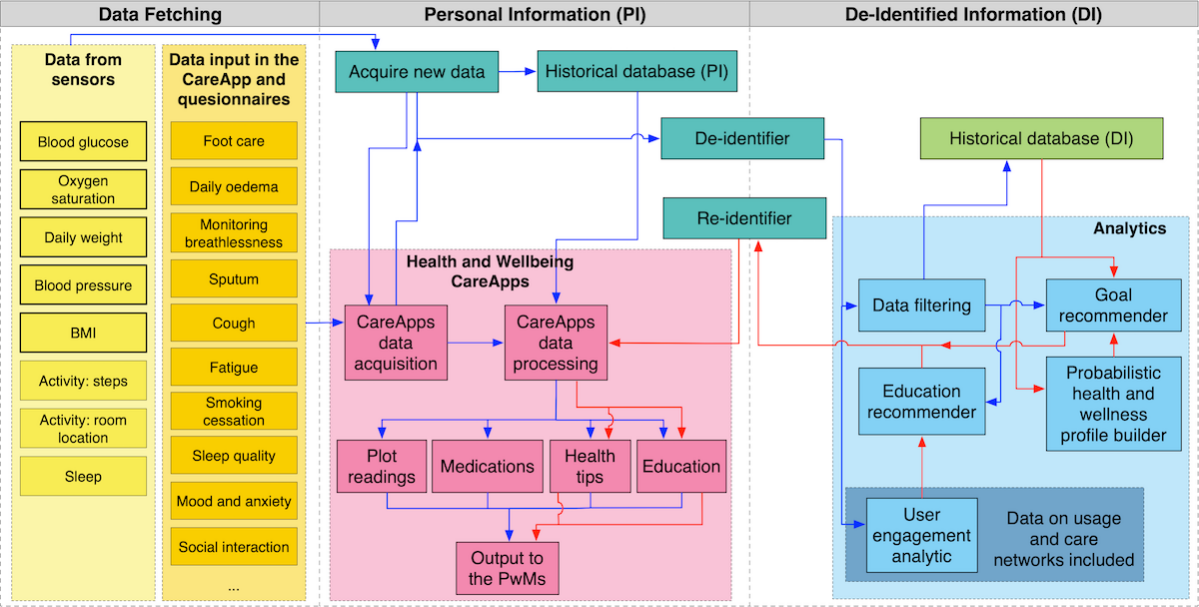
Data flow diagram implemented in ProACT. Blue arrows: data exchanges that occur with any new data input; red arrows: data exchanges happening at regular intervals, depending on the analytic. PwM: person with multimorbidity.

From a logical point of view, KITE acts as a gate for all communications, modeled in an asynchronous fashion to ease maintenance and monitoring. Figure 12 shows the data flow diagram within ProACT (for simplification, CareAnalytics are represented with one box only; however, they might be implemented as a set of simpler subcomponents). The diagram is divided into three main areas: (1) data fetching, (2) personal information, and (3) deidentified information. The left section lists all the data collected and pushed into the monitoring system managed by CABIE+. The data are deidentified and sent to the analytics that deal with deidentified information (right section). Although personal information data are stored on CABIE+, the deidentified information data (used by analytics) are stored in KITE.

Within KITE, CareAnalytics have two main purposes: making recommendations to people with multimorbidity (eg, by suggesting a goal or picking the most appropriate training material) and augmenting data (eg, creating aggregation or person-centered results).

### CareAnalytics

A *CareAnalytic* in ProACT is defined as a contextually aware procedure or algorithm that can detect and react to patterns in current or historic data available to ProACT systems. The current catalog of CareAnalytics in ProACT is as follows:

- *Data Cleaner*: this analytic classifies the different types of data in terms of quality (or reliability). In particular, the analytics that require vitals as input work on the results of the data cleaner instead of working on raw data. In addition, it calculates thresholds (ranges considered as normal) for the vitals that serve as an input for the *Education Recommender* (described below).
- *Goal Recommender*: this analytic supports people with multimorbidity in setting the weekly activity goals. The user can choose to set a goal in terms of distance, steps, or minutes spent walking. To suggest a goal value, the recommender considers the person with multimorbidity’s physical activity over the previous week and whether they have met their previous goals. It also considers physical activity guidelines for older adults and adults with chronic conditions [48] to avoid giving major leaps in recommendations. Finally, a maximum threshold (18,000 steps per week) was set as the total recommendation to ensure that the levels of activity were not too high for patients with chronic conditions and older adults [48].
- *Education Recommender*: this analytic facilitates the presentation of personalized educational and training materials within the CareApp. It uses thresholds on vitals; therefore, if any parameter has reached a value outside the normal range, then the person with multimorbidity will be presented with relevant educational material.
- *User Engagement Analyser*: this analytic tracks the person with multimorbidity’s use of sensing devices and the ProACT CareApp.
- *Probabilistic Health and Wellness Profiler*: this is a probabilistic model of the person with multimorbidity describing them through several dimensions. Specifically, a categorical Bayesian network was learned using the open The Irish Longitudinal Study on Ageing (TILDA) data set. The TILDA data set holds data on 8504 individuals aged 50 years and above [49]. Variables were selected to cover several dimensions relevant to the conditions being monitored by ProACT and other relevant parameters such as vitals (eg, symptoms such as blood pressure), assessments (eg, nonsensor data such as fear of falling), self-reported wellness (eg, sleep quality), behaviors (eg, steps or physical activity), demographics and conditions (including the ProACT conditions COPD, CHF, CHD, and diabetes), and relevant comorbidities, such as additional chronic conditions, depression, and anxiety. The main goal of the model is to provide a comprehensive overview for a specific person with multimorbidity and their health and well-being states across different dimensions. Furthermore, the Bayesian network model could be used to infer the level of a missing variable based on the available measurements. For instance, a possible output would be the probability of a low physical activity score for a male aged 70-75 years with high blood pressure and diabetes. Some or all of these outputs can be used by other analytics. Further information can be found in Deparis et al [50].

During the ProACT PoC trial, detailed below, the *Data Cleaner*, *Goal Recommender*, and *User Engagement Analyser CareAnalytics* were implemented and used within the ProACT platform. The development of the *Health and Wellness Profiler* and the *Education Recommender* only occurred as data became available during the trial to help build and validate these analytics, and as such these were not present within the ProACT platform during the trial. However, the goal is to integrate these into future versions of the platform to further enhance personalization and multimorbidity management.

## Methods

### Inclusion Criteria and Recruitment

Trials occurred in Ireland and Belgium. The inclusion criteria for people with multimorbidity were that they were aged >65 years and had a diagnosis of two or more of the following diseases: diabetes, COPD, CHD, and CHF. It should be noted that 1 participant in Belgium was aged 60 years. This participant had initially recorded an incorrect date of birth on the screening documentation, and this was not discovered until after the participant had already begun the trial. This person was excluded from the core analysis presented in this paper. However, a separate analysis of their data was conducted, and the outcomes reflected those participants aged 65 years and above. Participants were recruited from a number of sources, including social groups for older adults, condition support groups, social media, radio and local newspaper advertising, formal care organizations, health care professionals, pharmacists, and living lab agencies. People with multimorbidity could also nominate up to 5 people to be part of their care network (including informal and formal carers and health care professionals). A total of 73 care network participants consented to participate. However, this paper focuses solely on the person with multimorbidity participants.

### Study Design

**Figure 13:**
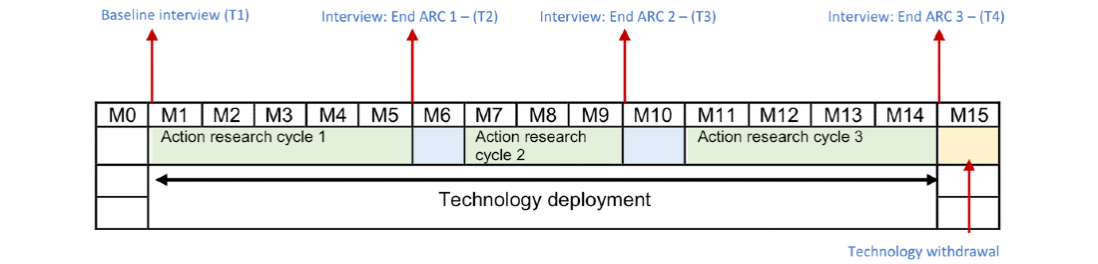
Study timeline across action research cycles. ARC: action research cycle.

The study was a PoC trial, which used an action research design, to allow for continuous feedback from participants and refinement of the ProACT platform throughout the trial. There were 3 action research cycles (ARCs) and 4 time points (T1-T4) of data collection, involving collection of questionnaire data, semistructured interviews, and usability testing (Figure 13). Refinements were made to the ProACT platform based on the feedback from participants at the end of each ARC. The same study design and methodologies were used across Ireland and Belgium to ensure consistency and comparability. The specific objectives were as follows:

- Evaluate the *usability and acceptability* of the ProACT CareApp and devices
- Evaluate *user adoption and satisfaction* with the technology and services
- Evaluate *experiences of using* ProACT

In this paper, we focus on the findings from studies that relate to engagement with the platform and usability.

### Procedures

Each participant was provided with an iPad, a smart watch to monitor sleep and activity, a digital weight scale, and a digital blood pressure cuff. Those with diabetes were provided with a blood glucose monitor (glucometer), whereas those with COPD were provided with a pulse oximeter to measure oxygen saturation levels. During the initial visit to the participant’s home, the devices were set up by the researcher, and training was provided on using the devices. A second visit, approximately 1 week later, introduced the ProACT CareApp to the participants, and further training was provided. Each of the participants received a detailed paper-based training manual, while training videos on how to use the devices and CareApp were also available in the *Tips* section of the CareApp. The participants were asked to use the ProACT platform as they wished to evaluate how ProACT would fit into their lives naturally. During the trial, data from the participants were monitored by a clinical triage service in each country, appointed through a tendering process. The triage staff determined a protocol for alerts and escalation procedures in the case of an alert. The alerts were set within the SIMS triage platform described above. These were initially set at a global level (eg, the same blood glucose thresholds were set for all diabetic participants) and as the trial progressed, these could be individualized based on a person’s normal range or input from their health care professional. The triage staff responded to alerts between 9 AM and 5 PM, Monday-Friday (hours were limited because of project budget) and made monthly check-in phone calls. Participants were made aware of the hours of triage, both through their project information sheet and a weekly pop-up message in the CareApp. Participants also had a helpdesk number they could call if they experienced any technical issues or wanted to request further training. A researcher was available to answer calls between 9 AM and 5 PM, Monday-Friday. Outside these hours, the participants could leave a message.

### Data Collection and Analysis

The full study protocol, including details on all data collected during the trial and full analysis procedures, can be found in Dinsmore et al [51]. For the purposes of this study, data collection comprised interview data and questionnaire data on usability and user burden at each time point, which coincided with the end of each ARC. These data were collected at the participants’ homes. Interview protocols at time point T1 covered motivations and expectations, whereas interviews at later time points covered a range of topics with respect to participants’ experiences in terms of using the technology, benefits and challenges, their self-management practices, and care network support. Interviews were audio recorded and transcribed verbatim. A semantic thematic analysis [52] of these transcripts was then conducted using NVivo for Mac (version 11) in Ireland and MAXQDA in Belgium. A selection of transcripts was coded by 2 researchers to ensure a thorough iterative identification of a wide range of semantic themes. Initial broad coding was performed to identify the themes of interest, as covered within the interview protocols. Within these broader themes, an iterative thematic analysis was conducted to uncover the subthemes. Themes with respect to engagement are presented in this paper, whereas other themes (such as those relating to the self-management journey, experiences with the technology, behavioral change, and collaboration with the care network) are being submitted for publication elsewhere. Data on engagement with the system were logged through the ProACT platform, and metrics analyzed included the number of symptom readings per day and engagement with different sections of the CareApp.

Although additional data were collected during the trial, such as interview data with care network participants and triage staff, as well as the symptom and well-being data collected through the ProACT platform, the analysis of such data is outside the scope of this paper and will be published elsewhere.

### Ethical Considerations

Ethical approval was received from 3 ethical committees in Ireland and 4 in Belgium. All procedures were in line with the General Data Protection Regulation for research projects, with the platform and trial methods and procedures undergoing data protection impact assessments in both countries. Written informed consent was obtained on an individual basis from participants in accordance with legal and ethical guidelines in each trial region, following a careful explanation of the study and provision of patient information and informed consent forms in plain language. All participants were informed of their right to withdraw from the study at any point without having to provide a reason for this.

## Results

### Overview

In total, 120 people with multimorbidity consented to participate, 60 in Ireland and 60 in Belgium. In Ireland, the average age of the participants was 74.23 (SD 6.4) years, and 60% (36/60) were male. In Belgium, the average age of the participants was 73.61 (SD 6.49) years, and the participants were predominantly male (43/60, 72%). Additional demographic data are presented in Table S1 of Multimedia Appendix 1. In this section, we present findings with respect to engagement with the ProACT platform, usability, and user burden and outline findings from the interviews with person with multimorbidity participants across the different time points that relate to engagement with ProACT. In particular, we focus on motivations to engage along with facilitators and barriers to engagement. At the end of the participant quotes, we identified the participant with the legend (ID, gender, age, inclusion conditions, time point, and country).

### Engagement With ProACT

Over the course of the 12-month trial, the majority of participants remained engaged with ProACT. By the end of the trial, 3 participants had died in Ireland and 8 had withdrawn, resulting in 49 participants in Ireland completing the trial. In Belgium, 16 people withdrew, resulting in 44 participants completing the trial. Exit interviews were conducted with a subset of those who withdrew early and who consented to this. The reasons for withdrawal are discussed below in the *Barriers to Engagement* section.

**Figure 14:**
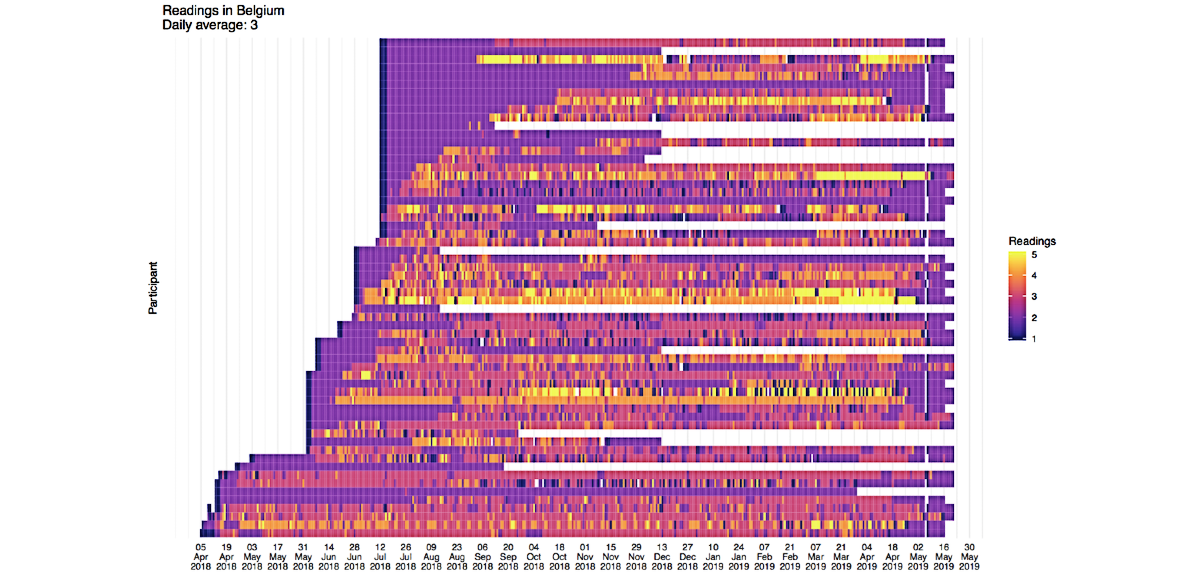
User engagement in Belgium—daily symptom readings.

**Figure 15:**
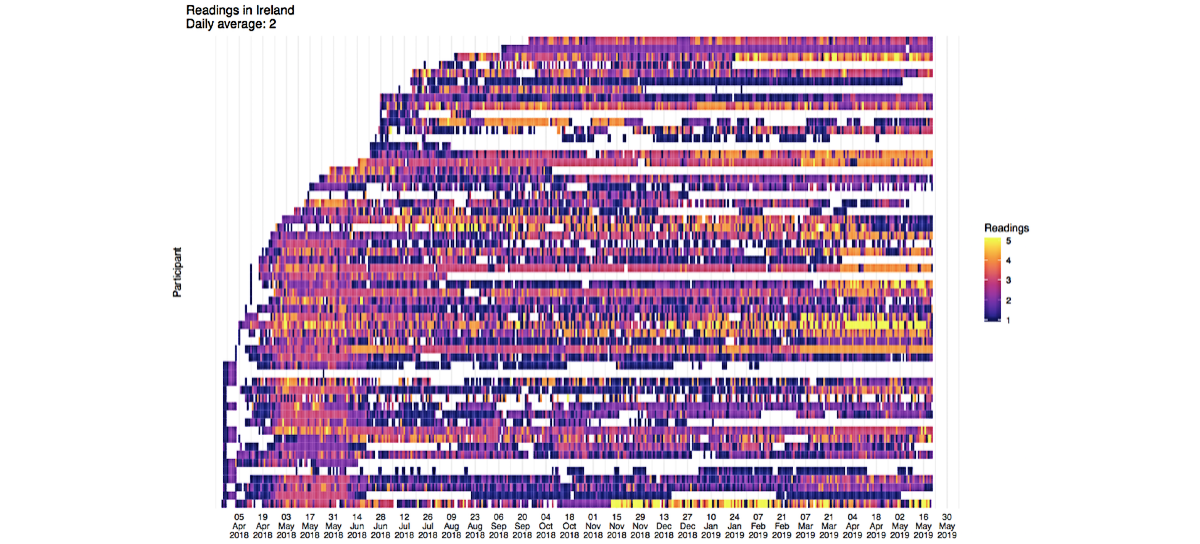
User engagement in Ireland—daily symptom readings.

Engagement with the sensor devices (used to record key symptom data) and ProACT CareApp were measured through the platform. The heat maps in Figures 14 and 15 illustrate high levels of engagement with the ProACT devices for measuring key symptoms and well-being parameters, by the majority of participants in Belgium and Ireland, respectively. The horizontal bars of each graph depict one participant, with the start of the bar indicating their recruitment to the trial and the end of the bar indicating their exit from the trial. White bars indicate dropouts or those who died. During the trial, there was an average of 40 (SD 7.6) users in Ireland and 43 (SD 16.6) users in Belgium, taking measurements on a daily basis. The maximum number of participants recorded taking measurements on any day during the trial was 48 in Ireland and 60 in Belgium. Participants had an average of 2 daily readings in Ireland and three daily readings in Belgium.

**Figure 16:**
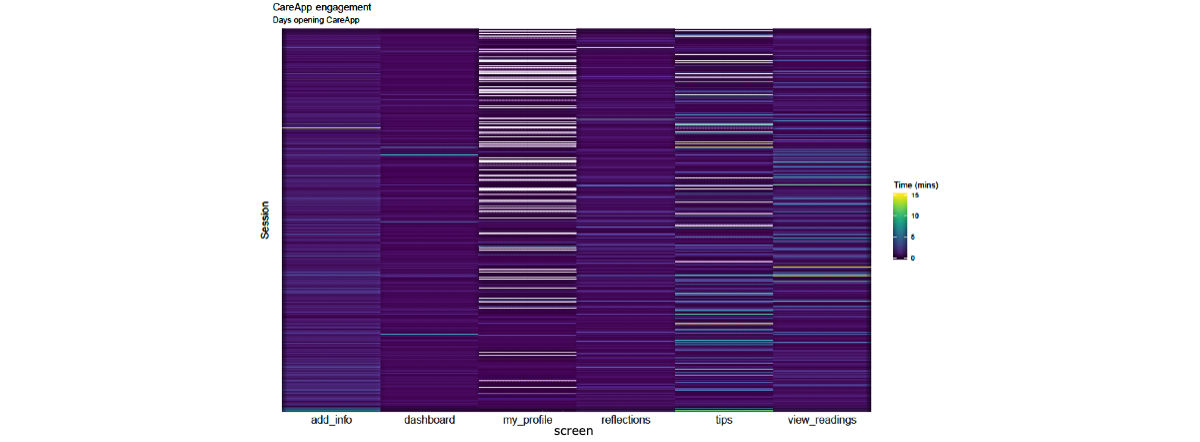
Heatmap of CareApp section visits across the trial period.

Figure 16 shows how the ProACT CareApp was used across all trial participants: each row is a session and each cell is colored depending on the time spent on each section of the CareApp (the sections can be seen in the bottom menu in Figures 3-5). White cells are sessions with no visits to the correspondent section. With this in mind, *My Profile* and *Tips* were less visited sections. The rest of the sections are quite dense in the graph, which means that they have been frequently visited. More green cells are visible in *View Readings*, which means that participants spent more time checking their vitals than entering data or looking at their *Dashboard* (Figure 3). Furthermore, *Tips*, despite being a less visited section, has a significant number of green cells, which is expected as participants would likely spend more time here, viewing relevant educational videos or reading health tips.

**Figure 17:**
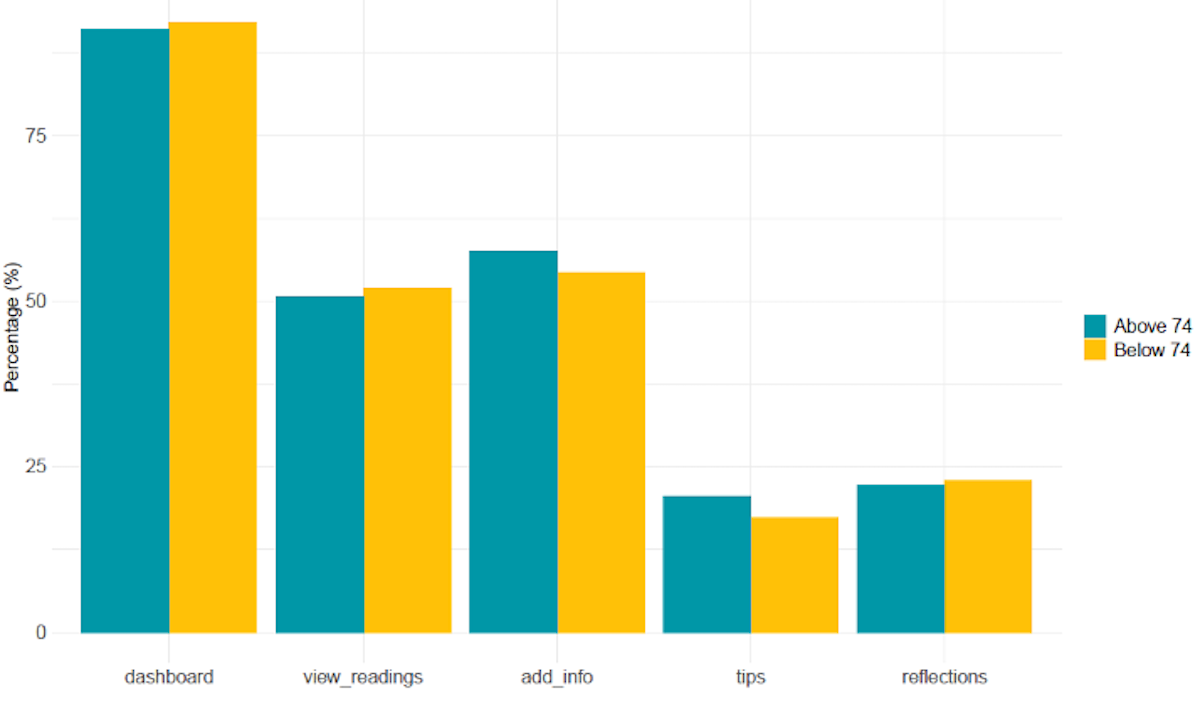
Percentage of time participants engaged with different sections of the CareApp across the trial period, categorized by age group.

Figure 17 shows the percentage of time, on average, that trial participants spent in different sections of the CareApp. No significant difference was recorded for those aged less than 74 years compared with those aged over 74 years across the different sections of the CareApp. When a user opens the CareApp, they land on the *Dashboard*, which accounts for the highest percentage of section visits. The *View Readings* (where participants could see an overview of all their health and well-being data) and *Add Info* sections (where participants could answer the daily questions or add a manual symptom reading) were the most frequently visited sections by both age groups. Participants navigated to the *Tips* section less often. This may be because the *Tips* were used mostly at the beginning of the trial, as participants were learning about their conditions and self-management. Future work will examine this issue further. Similarly, the reflection feature (Figure 4), which asks participants if a particular daily reading is high or low for them, was not used as much as other sections of the CareApp, and our data show that its use declined over time.

Finally, the *Goal Recommender* was implemented within the platform for the final 8 weeks of the trial, primarily to test whether it worked as expected. Approximately half of the participants (53/96, 55%) set at least 1 activity goal during this period. Almost 75% (39/53) of participants set their goals in steps rather than meters or minutes. Furthermore, participants felt comfortable with the metric chosen—just 3 changed it over the course of the 8 weeks (from distance to steps).

### Usability and User Burden of ProACT

The participants completed the system usability scale (SUS) at T2, T3, and T4. The SUS is a 10-item questionnaire and is a quick and reliable tool for measuring the usability of both hardware and software products [53]. SUS scores range from 0 to 100. A value above 68 indicates that the system is usable. Table 1 indicates that SUS scores during the trial over time points T2, T3, and T4 remained relatively the same in Ireland and Belgium and above the threshold of 68, which indicates that participants considered ProACT to be usable. User burden was also measured at T2, T3, and T4 using the user burden questionnaire that measures the burden across six constructs, including difficulty of use, physical, time and social, mental and emotional, privacy, and financial [54]. No significant increases in burden were experienced over time.

**Table 1:** Usability and user burden scores across time points in Ireland and Belgium.

		Time points, mean (SD)		
		T2	T3	T4
**Usability**				
	Ireland	74.6 (14.6)	72.4 (14.5)	75.6 (14.7)
	Belgium	78.1 (17.0)	74.9 (16.8)	75.5 (20.8)
**User burden**				
	Ireland	4.7 (4.0)	5.0 (4.4)	5.5 (4.6)
	Belgium	3.2 (3.1)	5.2 (5.1)	5.8 (6.6)

Participant interviews provided insights into possible usability and burden issues associated with using the platform. The SUS did not distinguish between the ProACT CareApp and symptom-monitoring devices. However, participant feedback suggests that the majority of usability issues lay with using the devices, in particular the blood pressure monitor and the glucometer, which was a source of frustration for some participants:

I just stopped taking the blood pressure measurements. Because all the equipment wasn’t working properly. In that it wasn’t connecting to the iPad. There was one morning I timed it and it was only after spending half an hour connecting the thing on the screen.

P009, male, 71 years, COPD+CHD+Diabetes, T3, Ireland

Questions about accuracy were also noted: “Look last Friday it did not register sleep, why not?” [P106, male, 70 years, diabetes+CHD+CHF, T2, Belgium]. P103 (female, 79 years, diabetes+CHD+CHF, T2, Belgium) who had mobility issues stated that her limited steps do not always register.

Despite several reported device issues, participants felt that the platform was usable:

The app itself I think is very good. And I think it’s helpful to get people to focus on a small number of things. That are key to their, you know their health. To also have a system that was easy to use, it is easy to use.

P015, male, 82 years, diabetes+CHD, T3, Ireland

Everything links in quickly enough...And I don’t have a problem, there’s no delay or whatever in it. It’s straightforward. I think the equipment is excellent.

P012, male, 67 years, diabetes+CHD, T3, Ireland

### Motivations to Engage

#### Overview

Two primary subthemes emerged with respect to motivation to engage at T1, which remained present at T2, T3, and T4 as reasons for continued engagement with ProACT:

- Improving self-management
- Improvement of health and well-being

#### Improving Self-management

At the start of the trial, improving self-management was noted by the majority of participants as the reason they wanted to engage with ProACT:

I haven’t focused sharply enough [on health] and this is where I think this programme maybe will give me a bit of a kick in the backside, where it’s very in your face and very visual and you know, just to see like say the blood pressure or the blood sugar readings.

P033, male, 65 years, diabetes+CHD, T1, Ireland

The participants felt that improved self-management would be achieved by gaining insights into their health from actively self-managing and seeing results, learning about conditions, learning what to avoid, and learning what to do if symptoms are changing.

At the start of the trial, a number of participants discussed how they hoped using ProACT would lead to increased confidence both in terms of managing their health and engaging with activity:

You know, seeing the results, it might give more confidence...I’d like to be able to have a round of golf and also I’d love to get back and play an odd set of tennis.

P052, 78 years, male, diabetes+CHD, T1, Ireland

P052, like many participants before beginning the trial, was fearful of undertaking physical activity in case it exacerbated their conditions. Interview data from later time points in the trial show the participants became more empowered and confident in their self-management, as they used the ProACT CareApp to reflect on and compare data from a range of devices, in 1 place, across multiple symptom and well-being readings:

[The technology] gives you confidence, to be honest with you. And I quite like being able to take my own blood pressure and stats, so as I’m still in control.

P031, male, 70 years, CHF+CHD, T2, Ireland

P115 spoke of her fear before the trial, as an exacerbation could happen at any time:

I was living on a timebomb. Now, I do not live on any different timebomb than anyone else.

P115, female, 73 years, CHF+CHD, T3, Belgium

You are not as frightened because you know your readings and everything like that.

P036, male, 72 years, COPD+CHD, T4, Ireland

This was partly linked to higher levels of awareness:

Well, I suppose by being a bit more aware and a bit more in control, it’s a positive thing and I suppose you have a bit more confidence as a consequence.

P033, male, 65 years, diabetes+CHD, T2, Ireland

Participants also reported making sense of how their actions affected their health, giving insight into what behaviors they could moderate or change to improve their various health readings:

Well it’s telling me what’s going on I suppose, I’ve a better idea myself and can relate the two together. If I haven’t had activity, I can see how that is affecting blood sugars. I can see the relationship between the two, it’s becoming clearer.

P012, male, 67 years, diabetes+CHD, T3, Ireland

Related to this, participants reported taking action based on their insights across their conditions and well-being, including changing their behaviors to improve their health readings. Participants also reported taking action with respect to their use of health care and interactions with health care professionals:

It is one of the reasons, through the use, that I went to my GP. That I said look, I am a bit worried. And then we looked for a solution and different medication and such. And that has changed due to [ProACT].

P71, male, 69 years, COPD+CHD, T2, Belgium

Notably, some participants reported going to the doctor less as they had more confidence from knowing their readings:

When I get an attack, the COPD or whatever flares, I can recognise [now] whether I need an antibiotic or not...Whereas before [using the technology], as soon as my breathing sort of laboured I’d be at the doctor.

P045, 74 years, female, diabetes+COPD, T4, Ireland

Many participants experienced benefits from such actions, which motivated engagement with the ProACT platform:

It’s a whole health awareness...That’s what [the technology] has created. Before this, I knew I needed to look after my health, but nobody does, and I got this system, now it’s become a matter of routine.

P053, male, 71 years, diabetes+CHD, T3, Ireland

For P034, who reported struggling with mental health challenges, it was especially reassuring to see mood changes including periods of positive mood:

Oh, that was the highlight of my week, looking back on how I was doing...If I saw my graph was going up, and I wasn’t as anxious yesterday as I was before, that would give me a bit of a boost.

P034, 67 years, male, COPD+CHF, T4, Ireland

#### Improving Health and Well-being

At T1, many participants expressed hope that engaging with ProACT and improving their self-management skills would in turn lead to improved health and a better quality of life, and this was the case for a number of participants:

My health has improved immensely...I’ve probably got the AFib [atrial fibrillation] under control...But, as I say, I’ve lost weight and I feel much better in myself. Mentally, physically, every way.

P031, male, 70 years, CHF+CHD, T3, Ireland

Indeed, throughout the trial, there were a number of critical clinical outcomes reported by participants in both Ireland and Belgium, where engagement with ProACT exerted a clear impact on a health outcome or directly influenced a change in treatment or care for the participant:

I’ve been in with my GP, he’s increasing the medication. And we’re still not there with it, so it may need to be tweaked further. So, without this system I might’ve been, you know, pretty oblivious to the fact that there is an issue there you know.

P033, male, 65 years, diabetes+CHD, T3, Ireland

Participants reported *symptoms stabilizing* as a result of implementing self-management practices:

I mean on average they [blood glucose readings] were 14 or 15 [14 mmol is threshold for a high alert], before this research started. And that was continuous. Now it’s halved. Because I’m focused on improving it.

P014, male, 70 years, diabetes+CHD, T3, Ireland

Changes in food and diet were also widely reported, as well as increased activity levels, particularly as participants began to see the links between their activity and symptoms:

Well, the whole thing for me really is trying to get my weight down and keep my blood sugars down. And it’s more stable...It’s [ProACT] done everything really, it’s gotten my exercise levels up because it focused me on it. It’s got my blood sugars back in order.

P048, male, 67 years, diabetes+CHD, T2, Ireland

When I started [ProACT] up to now, I feel fitter...It is only 4000 steps, but I used to do maybe like 300 steps a day.

P111, male, 76 years, diabetes+CHD+COPD, T3, Belgium

Although participants in Belgium reported experiencing stabilization of symptoms and improvements in terms of activity and weight, few participants explicitly stated that they thought their health had improved. Most of the perceived health situations remained stable, except for those who endured health setbacks because of their illnesses or unfortunate incidents (eg, P117 had a fall and P81 had a car accident). However, 1 participant linked his engagement with the technology and the triage service with his continued ability to live alone:

They [family] were planning on doing that last year [sending him to a nursing home], now I do not have to, because I can make my own plan, because I can control myself.

P74, male, 72 years, diabetes+CHD+CHF, T3, Belgium

When participants attributed potential changes in health to using the platform, they were related to changes in attitude or behavior.

### Potential Facilitators of Engagement

An important element of the ProACT platform, which was implemented during the trial, was the service provided alongside platform usage. A large theme that emerged across T2, T3, and T4 was *Service and Support*, which had 2 elements, namely, the triage service and the technical helpdesk. The triage service was one of the most commonly discussed topics by ProACT users in Ireland and Belgium**.** The majority of participants commented on the *reassurance* of having their readings monitored by the triage service. Most found it beneficial to know someone was looking out for them behind the scenes and making sure their readings were within a normal range:

The fact that...I knew someone was keeping an eye on it. And if that something was going out of kilter [range] that I’d get a nudge. And that’s all I’d need...I know there’s a nurse there who’s looking at these figures.

P015, male, 82 years, Diabetes+CHD, T3, Ireland

Participants also noted how the triage nurses acted as a *backup* if they were to miss something, which gave further peace of mind:

It has given my wife a feeling of reassurance. People are keeping an eye on me. For me...it is also a reassurance.

P81, male, 68 years, CHD+CHF, T4, Belgium

As noted above, a number of participants reported clinical outcomes during the trial, and many of these were a result of triage intervention:

They [triage] had suggested maybe to go and get my cholesterol checked and my cholesterol was a bit high. It had been going a bit high and then my medication was changed, and it was as a result of ProACT.

P039, female, 66 years, COPD+CHD, T4, Ireland

[Triage nurse] told me get her into a hospital straight away otherwise you’re going to have a body on your hands. Bring her out to the hospital and tell them the way she is. And I did that, and they kept her in...Yeah she [nurse] wanted to send an ambulance.

Husband speaking during T3 interview for P018, female, 73 years, diabetes+CHD, T3, Ireland

Social connection and personalized care were also identified as a subtheme within the triage service theme, whereby users felt that the triage nurses truly cared about their well-being. The majority of participants commented on how the nurses took the time to chat to them, enquire about their health, and found the phone calls to be a very pleasant experience: “What I think is so good about it, is that you have the feeling that someone thinks it is important what I do.” [P101, male, 72 years, CHF+CHD, T3, Belgium]. Participants also appreciated the continuity of care and that the nurses always followed up on any issues, something many participants felt was lacking with their usual health care providers.

The research teams also operated a technical helpdesk that participants could call (Monday-Friday, 9 AM-5 PM) if they were experiencing any difficulties using the technology. When a participant called the helpdesk, the researcher attempted to troubleshoot with the participant over the phone. If this was not possible, then a researcher visited the participant to ensure that the issue was resolved. The responsiveness of this service was an important element in ensuring that participants could remain technically engaged with the ProACT platform and provide a fast and meaningful response to technical barriers encountered by participants, which might otherwise have resulted in disengagement:

I was helped along every time...[researchers] arrived straight away or as soon as possible to sort it out for me.

P043, female, 77 years, COPD+CHF, T4, Ireland

To offer context to the necessity of the helpdesk, there were a total of 355 calls to the helpdesk in Ireland over the course of the trial (helpdesk calls were not tracked in Belgium). Of these, 22% (78/355) were administrative calls, such as participants informing the team of an upcoming holiday or requesting clarification on interview dates. The remaining 78% (277/355) were related to tech support. Of these, 76 calls were requests for peripherals (eg, batteries for the blood pressure cuff and additional testing strips for the blood glucose monitor), 121 were for devices not working (eg, a blood pressure cuff that would not turn on), 50 related to data syncing issues (eg, if participants did not see their readings appear in the ProACT CareApp), 31 related to data accuracy concerns (eg, if a participant’s own blood glucose monitor had a different reading to the digital one provided with ProACT), and 29 involved pairing or connection problems (eg, accidentally turning off Bluetooth).

### Barriers to Engagement

Although the majority of participants learned to master the ProACT technology over time, technical issues were a barrier that ultimately led to some participants withdrawing from the trial. For P007 and P037 in Ireland, the reasons for withdrawal were *frustration with the devices*. Both found the devices and apps complicated to use: “It was just too hard to do because you couldn’t in the first place take the blood pressure anyway because it wasn’t working.” [P037, female, 82 years, COPD+CHD, Ireland]. Although care was taken to address these issues, for example, by replacing devices and providing further training, the initial experience for these participants resulted in a negative perception of the technology, which led to their disengagement. Related to this was a lack of trust in the readings. Some participants, including P93 and P105 in Belgium, found that the digital glucometer provided for the trial gave higher readings than their own personal glucometer, which caused them to distrust data from the trial device.

The second most common reason for withdrawing from the trial involved the complexity of the participants’ conditions. For some, monitoring with ProACT felt like an added burden. Three participants in Ireland who withdrew did so as they felt they had enough to deal with in managing their conditions or with health complications which had arisen since the start of the trial. This was expressed by an informal carer for P007: “I think P007 has enough to contend with at the moment” [IC for P007, female, 78 years, diabetes+CHD, Ireland] and by P037 who felt under pressure to generate readings:

Well I was conscious of how much I was walking, you know and I’d walk up and down to the shed a whole lot of times if I wasn’t out. So that was it, but I couldn’t stick that for a year.

P037, female, 82 years, COPD+CHD, IE

P007’s informal carer noted that readings generated concern: “Sometimes it was a little bit scary when you were getting those readings.” P105 (76 years, female, diabetes+COPD+CHD, Belgium) stated that she experienced an extra burden by being confronted by some of the information provided by ProACT. She said that as a patient with diabetes, she already needed to be aware of a lot and that the pressure she felt from seeing the additional readings, such as activity, was experienced as too much of an extra burden.

## Discussion

### Principal Findings

This paper has presented the ProACT platform alongside findings with respect to engagement and usability from a 1-year PoC trial of the platform with 120 older people with multimorbidity across 2 EU countries. The findings demonstrate that the majority of participants actively engaged with the platform over the full duration of the trial, taking on average between 2 and 3 readings every day and engaging with self-reporting and their data in the CareApp. The qualitative data indicate that participants were motivated to engage as they found value and benefit in using the platform, including improving their self-management, gaining confidence, and experiencing health and well-being benefits. The results also indicate that participants found the platform usable and of low burden. Our study makes two primary contributions to the literature. First, we describe a novel, comprehensive digital platform, including CareApps, participant management, clinical triage, and analytics, and outline how it has been designed to support the self-management of multimorbidity. A recent systematic review highlighted that such platforms are lacking, and those that exist only target single disease management [29]. Second, we demonstrated that older adults were actively engaged with the platform over a 1-year period and we presented qualitative data to understand the possible reasons for this result. Textbox 1 summarizes the potential factors that may have enhanced engagement, which are elaborated upon in the remainder of this section.

**Likely impact of ProACT components on engagement.**

**Outcomes**

- Engaging with symptom and well-being monitoring
- Engaging with reviewing data and acting on it

**Potential Factors That Enhanced Engagement**

- User-centered design (understanding needs and requirements)
- Usability of app
- All data on 1 platform
- Benefits experienced because of monitoring
- Technical support when needed, including face-to-face training during deployment and technical training via the application supplemented by paper-based materials
- Clinical oversight and support provided by triage nurses

Recently, researchers of human-computer interaction have begun to explore how technology can support the self-management of multimorbidity [18,21,22,24,55]. The majority of this research has focused on suggesting design requirements for technology, including understanding the requirements of people with multimorbidity and those who care for them [21-23,55], supporting communication about values with people with multimorbidity and health care professionals [27], managing health care conflicts [24], and medication management [20,28]. Research has also suggested potential reasons for those with multimorbidity not engaging with technology-supported self-management (including self-tracking being experienced as *work* and perceptions that health care professionals are not interested in self-tracked data) [18]. Our work in designing and developing the ProACT platform occurred before much of this recent research was published, with the trial beginning in April 2018. However, there are parallels in terms of design suggestions for multimorbidity management. Ancker et al [18] stated that technologies for multimorbidity self-management will only be successful if they do not place further inconvenience or burden on the person. Indeed, earlier phases of our work, to understand user needs, highlighted how consuming it is living with and managing multimorbidity [21]. This was one of the primary drivers for designing a single platform for multiple self-management tasks, which includes features and analytics to help people with multimorbidity prioritize their conditions (eg, by highlighting on the dashboard *flower* those currently needing attention; Figure 3).

Caldeira et al [24] suggested three design guidelines for technology to support multimorbidity management: promoting conflict awareness (between disease and self-management approaches), supporting conflict resolution (to the most relevant approaches depending on symptom changes), and promoting patient expertise (to enact the approaches needed), which were considered while designing the ProACT platform. Considering patient expertise, the authors suggest that providing detailed health information and education can facilitate learning and contribute to expertise [24]. Our earlier findings also highlighted the lack of support people with multimorbidity receive in self-management [21], which identified the need for educational material, appropriate training on using self-management technology, and a triage service that would not only respond to symptom alerts but also motivate, support, and reinforce education presented through CareApp. People with multimorbidity in our study reported more awareness and increased confidence with respect to self-management, demonstrated the knowledge of their conditions, and what can impact them. With respect to designing for conflicts, CareAnalytics within ProACT have been designed to support the recognition and resolution of conflicts. For example, the Health and Wellness Profiler computes a probabilistic description of the person with multimorbidity, which is particularly important for multimorbidity, and all conditions can be considered while assessing the risk associated with the other variables in the model. For instance, the estimated probability of a given physical activity level is not the same if the person with multimorbidity has a fear of falling in addition to COPD. Using an automated goal recommender that considered individual patterns related to the performance of a given behavior (eg, goal achievement, optimal levels of activity), disease, symptoms, and usage of the recommendation feature, contribute to more personalized and adaptive digital health interventions. The goal recommender was only implemented for 8 weeks during the trial and was focused solely on physical activity goals. Future work with respect to this feature and analytic will focus on a more holistic approach to goal setting to better support the self-management of multimorbidity, in line with findings identified in our previous research [36]. Similarly, the education recommender will ensure that any recommended content considers the person with multimorbidity’s condition and comorbidity profile as well as their current status to help them better understand potential conflicts and how to deal with them. With time, the artificial intelligence systems within ProACT are expected to learn from the data to provide more accurate recommendations and include additional parameters of personalization (eg, progress in other health behaviors, personalized education).

Despite this promising recent interest in understanding how to design technologies for the self-management of multimorbidity, there is little research on platforms that have actually been designed and implemented or evaluated for longitudinal periods. However, there is an abundance of research exploring how technology can support the management of single chronic diseases, including diabetes [56-58], COPD and related respiratory diseases [59], chronic kidney disease [60], and hypertension [61]. Our findings have many parallels with these studies. For example, Visser et al [58] explored the experiences of older adults using blood glucometers to monitor type 1 diabetes. The majority of participants reported that they sustained use of the device over time to know how much insulin was required before meals and because using the device gave a sense of safety and confidence. A greater confidence in practicing self-management was observed in our qualitative findings. The ability of the technology to provide novel insights into one’s conditions and support the prevention of exacerbations (keeping symptoms under control) was also identified in our study, with similar benefits reported by Tenendez et al [59] in their study of older people self-managing chronic respiratory disease. There are also some differences between our findings and those of others. Tenendez et al found that participants took a reactive approach to self-managing, including the monitoring of symptoms, only doing so when they felt unwell [59]. The authors suggested that the potential reasons for this are that monitoring chronic respiratory conditions can be overwhelming and make people more conscious of their disease. They also suggest that long-term daily self-monitoring can lack value for those with chronic respiratory conditions, something that has also been highlighted by others [62,63]. Our findings contradict this result, thus indicating that people did find value and benefit in engaging in monitoring and seeing their data, with additional perceived value because of the triage service providing backup and peace of mind.

Our findings with respect to engagement with the sensor devices and CareApp indicate a high level of engagement with symptom and well-being monitoring and review. However, research has noted the importance of *effective* engagement rather than simply sustained or *more* engagement, with effective engagement being defined as *sufficient engagement with the digital intervention to achieve intended outcomes* [30]. Intended outcomes, from the perspective of digital health interventions, often relate to changes in behavior and improved health and well-being. For the ProACT trial, the key behavior under examination was engagement with the platform for self-management. Given that the ProACT platform represents a novel technology, the objectives of the trial were to evaluate usage, usability, and experiences to allow for the refinement of the technology before a trial to determine its effectiveness. This has been deemed a necessary approach to digital health intervention development and evaluation [64,65]. For example, Klasnja et al [64] suggest that evaluations of novel technologies should focus on human-computer interaction outcomes, including efficacy evaluations of specific intervention strategies such as self-monitoring and gaining a deep understanding of user experiences. Engagement with interventions has also been described as a precondition for effectiveness [30]. Although effectiveness was not specifically measured in our study, the qualitative data indicated that participants changed their behaviors, including that they improved their self-management skills, got their symptoms under control, engaged in physical activity, experienced weight loss, and changed when and how they interacted with their health care professionals. Participants in Ireland also reported improvements in health. The analysis of the sensor and well-being data collected through the platform during the trial is currently underway, focusing particularly on symptom stabilization and physical activity to determine whether engagement with the intervention had a positive impact on these areas.

Previous research has indicated that the adoption of self-management technologies is limited [66]. Studies on the adoption of self-management technologies by older adults, particularly the oldest old, are lacking [67]. A number of studies have focused on conducting interviews, focus groups, or surveys with older adults to gauge interest in health technologies or barriers. Many of these reported negative attitudes. For example, Heart and Kalderon [32] found that older adults lacked interest in such technologies and perceived accessibility barriers. The authors concluded that such technologies would not be accepted by older adults unless they were very easy to use, useful, and there was support available to help with technical difficulties. The authors also suggested that when such factors are present, older adults can embrace self-management. On the basis of data from real-world engagement and participant feedback, our findings support this argument.

A number of possible reasons for sustained engagement during the ProACT trial have been identified. Throughout the trial, participants reported benefits, including improvements in health and well-being related to an increased ability to self-manage. Perceived benefit plays a role in technology acceptance and adoption [68,69] as well as in the activation of effective self-management behaviors [16]. Our findings are in line with those of a systematic review of studies examining older adults’ usage of technology to support chronic disease management, which found that participants experienced greater self-efficacy because of technology-supported self-management, which enabled improved communication with health care providers, including personalized feedback and support [17].

Yardley et al [30] state that “successful intervention design demands a user-centered and iterative approach to development, using mixed methods and in-depth qualitative research to progressively refine the intervention to meet user requirements.” A notable feature of the ProACT approach to designing and evaluating the platform was that it continually involved people with multimorbidity in a user-centered design process, including interviews, focus groups, co-design workshops, usability testing and evaluation activities, supporting regular, iterative updates to the ProACT platform. Our findings indicate that the majority of trial participants found the ProACT platform to be usable. Usability barriers are recognized within the literature as negatively impacting older adults’ adoption of and engagement with technology [70,71]. Our findings also indicated that the participants found using this technology exerted low burden. This is an important finding given that there is significant work associated with self-management of chronic diseases, particularly multiple diseases and comorbidities [41]. Although participants had self-management activities outside of using the platform and their interactions with triage, such as attending appointments and managing medications, which were not evaluated as a part of the trial, it is promising that engaging in symptom monitoring, review, and education did not result in a high level of burden for the majority of participants. The usability and burden findings, along with the engagement data, indicate that the extensive user-centered design process during the project lifecycle cannot be underestimated; without this effort, it is possible that users would not have sustained their use of ProACT.

Research has noted that using digital health platforms for self-management without human support can negatively impact engagement, resulting in dropout and nonusage attrition [30,72]. Human services, including the triage and helpdesk, provided alongside the ProACT technology platform, appeared to be an important factor in terms of contributing to sustained engagement. Participants received feedback and advice on their health and well-being from the triage nurses and experienced continuity of care. Many clinical outcomes were also precipitated by the triage staff. Ancker et al [18] suggested that participants in their study lacked motivation to self-manage, as they perceived that health care professionals were not interested in their data. Furthermore, participants using ProACT had access to a technical helpdesk for the study duration if they experienced any technical issues. As is evident from the Irish helpdesk data outlined above, this was a necessary service because across the trial period for 60 participants, 355 calls were made to the helpdesk, 277 of which were related to technical issues, primarily related to the third-party devices provided to participants for symptom monitoring. Indeed, frustration with the sensing devices, including a poor usability, lack of reliability, and mistrust of the data they produced, were reasons for participants withdrawing from the trial. Given the importance of such devices in health self-management, device providers should strive to ensure that they are usable and reliable. Other research on the abandonment of health tracking devices found reasons that included technology being too complicated, too complex to learn, or failing to help people reach their goals [73].

Future research should also examine the potential role and necessity of helpdesk and triage services over longitudinal periods of time, as the needs of people with multimorbidity, as well as their self-management and technical skills change. Research should examine at what point or points of a self-management journey human support adds value to a digital health intervention. For example, some research has shown that support for self-management is mostly needed when a person first begins using a digital health intervention [56] to ensure adoption. However, research into the type and level of ongoing support that might be required for self-managing with multiple, complex chronic conditions is lacking. This also needs to be considered within the context of aging, as many older adults will experience a decline in health or the diagnosis of additional conditions and comorbidities over time. However, it is important to note that the levels of triage and technical support required also have implications in terms of costs of service provision; for example, the ratio of nurses to patients that is required to provide an effective service. Hence, this aspect also needs to be further examined.

### Limitations

There are several limitations to this study. First, given the resource limitations, the version of the ProACT platform described in this paper and evaluated in the trial did not integrate all of the features required to fully support the self-management of multimorbidity, with development initially focused on symptom and well-being monitoring, education, setting physical activity goals, and data sharing. The inclusion of features such as medication management, care planning, and goal setting beyond physical activity would be required to fully address the challenges of the self-management of multiple conditions. However, the addition of such features could also lead to an additional technological burden, which could negatively impact engagement. The integration of CareAnalytics not currently integrated within the platform and further development of all analytics could help minimize burden while also enhancing the platform’s capability to handle recommendations that consider a person’s full condition profile. For example, future work aims to further enhance the goal recommender in terms of personalization by considering the person’s current health status, as indicated by the Health and Wellness Profiler analytic described above.

A further point with respect to the PoC study design (which was primarily to understand the feasibility of people with multimorbidity engaging with a digital health platform to self-manage their conditions) is that a control group was not included; therefore, it was not possible to assess the effectiveness of individual components of the ProACT platform and related services (access to triage and technical support). Future work will aim to examine the potential impact of these factors in more detail through controlled studies with people with multimorbidity. Implementing an iterative, user-centered PoC trial has helped justify the need for a large-scale controlled study. Therefore, we advocate that such an approach to designing and developing complex interventions for the management of multiple diseases should be considered before progressing toward large-scale studies.

### Conclusions

This work is the first to present a digital health platform designed specifically to support multimorbidity self-management for older adults, with results showing high levels of engagement and retention over a 12-month period. There are several strengths to this work. We present a comprehensive digital platform that outlines how various components support the management of multiple diseases. There was active engagement from participants across all stages of the design, development, and evaluation of the platform. The trial involved a large number of participants across 2 EU countries over a longitudinal period. Few studies on digital health solutions to support the self-management of chronic conditions have focused on older adults. Neither do they have such large numbers (especially at the PoC level) nor do they examine long-term usage. Furthermore, comparatively little research has examined the design and evaluation of digital health platforms for people with multimorbidity.

Planned future work includes updating the platform to further enhance the management of multimorbidity and a pragmatic randomized controlled trial to evaluate the effectiveness (including potential cost-effectiveness) of the platform and triage service on quality of life, health care utilization, and a range of other measures, which will begin in 2022.

## Appendix

Multimedia Appendix 1 Person with multimorbidity demographics.

## Abbreviations

ARC: action research cycle
CABIE+: context-aware brokering and inference engine
CHD: chronic heart disease
CHF: congestive heart failure
COPD: chronic obstructive pulmonary disorder
EU: European Union
KITE: Knowledge InTEgration
PoC: proof-of-concept
SIMS: subject information management system
SUS: system usability scale
TILDA: The Irish Longitudinal Study on Ageing
