# Supplementary material for: A Digital Platform to Support Self-management of Multiple Chronic Conditions (ProACT): Findings in Relation to Engagement During a One-Year Proof-of-Concept Trial
Source: J Med Internet Res. 2021 Dec 15;23(12):e22672. doi: 10.2196/22672 (PMC8717138; doi:10.2196/22672)
Supplement: Multimedia Appendix 1 [file jmir_v23i12e22672_app1.docx]

Multimedia Appendix 1. Person with multimorbidity demographics.

Table 1. Demographics of PwMs in Ireland and Belgium

|  | **Ireland** | **Belgium*** |
| --- | --- | --- |
| **Age**  mean, SD (range) | 74.23 ± 6.4 (65-92 years) | 73.61 ± 6.49. (60-91) |
| **Gender** N (%) | | |
| Male | 36 (60) | 43 (72) |
| Female | 24 (40) | 17 (28) |
| **Ireland Highest Educational level** N (%) | | |
| Some Primary | 3 (5.0) |  |
| Primary | 14 (23.3) |  |
| Secondary | 20 (34) |  |
| Junior Certificate | 10 (16.7) |  |
| Leaving Certificate | 10 (16.7) |  |
| Diploma / Cert | 12 (20) |  |
| Primary Degree | 1 (1) |  |
| Postgrad | 10 (17) |  |
| **Belgium – Highest Education Level** N (%) | | |
| None / Primary |  | 1 (2) |
| Lower Highschool |  | 14 (25) |
| Higher High school |  | 12 (21) |
| Higher Education |  | 14 (25) |
| University |  | 16 (28) |
| **Living status** N (%) | | |
| Lives alone | 25 (42) | 18 (31.6) |
| Lives with others | 35 (58) | 39 (68.4) |
| **Marital Status** N (%) | | |
| Married | 28 (46) | 36 (64) |
| Living with Partner | 4 (7) | 2 (3) |
| Widowed | 18 (30) | 7 (12) |
| Single (never married) | 4 (7) | 4 (7) |
| Separated | 4 (7) | 1 (2) |
| Divorced | 2 (3) | 5 (9) |
| **Employment Status** N (%) | | |
| Retired | 55 (92) | 52 (91) |
| Permanently Sick/Disabled |  | 1 (2) |
| Self-employed | 5 (8) |  |
| Other |  | 4 (7) |
| **Condition Profile** | | |
| CHF/CHD | 7 | 7 |
| CHF/CHD/COPD | 1 | 3 |
| COPD/CHD | 16 | 9 |
| COPD/CHF | 1 | 0 |
| COPD/CHD/Diabetes | 1 | 6 |
| Diabetes/CHD | 30 | 19 |
| Diabetes/CHF | 1 | 1 |
| Diabetes/COPD | 3 | 1 |
| Diabetes/CHF/CHD | 0 | 8 |
| Diabetes / CHF / CHD / COPD | 0 | 2 |

*N=57 Belgium, 3 participants dropped out before completing their demographics
